# Phosphate starvation of maize inhibits lateral root formation and alters gene expression in the lateral root primordium zone

**DOI:** 10.1186/1471-2229-12-89

**Published:** 2012-06-14

**Authors:** Zhaoxia Li, Changzheng Xu, Kunpeng Li, Shi Yan, Xun Qu, Juren Zhang

**Affiliations:** 1 School of Life Science, Shandong University, Jinan, Shandong, 250100, China; 2 Qilu Hospital, Shandong University, Jinan, 250012, China

**Keywords:** Maize, Phosphate starvation, Root development, Transcriptomic analysis

## Abstract

**Background:**

Phosphorus (P) is an essential macronutrient for all living organisms. Maize (*Zea mays*) is an important human food, animal feed and energy crop throughout the world, and enormous quantities of phosphate fertilizer are required for maize cultivation. Thus, it is important to improve the efficiency of the use of phosphate fertilizer for maize.

**Results:**

In this study, we analyzed the maize root response to phosphate starvation and performed a transcriptomic analysis of the 1.0-1.5 cm lateral root primordium zone. In the growth of plants, the root-to-shoot ratio (R/L) was reduced in both low-phosphate (LP) and sufficient-phosphate (SP) solutions, but the ratio (R/L) exhibited by the plants in the LP solution was higher than that of the SP plants. The growth of primary roots was slightly promoted after 6 days of phosphate starvation, whereas the numbers of lateral roots and lateral root primordia were significantly reduced, and these differences were increased when associated with the stress caused by phosphate starvation. Among the results of a transcriptomic analysis of the maize lateral root primordium zone, there were two highlights: 1) auxin signaling participated in the response and the modification of root morphology under low-phosphate conditions, which may occur via local concentration changes due to the biosynthesis and transport of auxin, and LOB domain proteins may be an intermediary between auxin signaling and root morphology; and 2) the observed retardation of lateral root development was the result of co-regulation of DNA replication, transcription, protein synthesis and degradation and cell growth.

**Conclusions:**

These results indicated that maize roots show a different growth pattern than *Arabidopsis* under low-phosphate conditions, as the latter species has been observed to halt primary root growth when the root tip comes into contact with low-phosphate media. Moreover, our findings enrich our understanding of plant responses to phosphate deficits and of root morphogenesis in maize.

## Background

Maize (*Zea mays*) is not only a key human food and animal feed crop throughout the world but also an important raw material for the food industry and energy production plants [1]. Low phosphate concentrations are frequently a constraint for maize growth and development, and therefore, enormous quantities of phosphate fertilizer are expended in maize cultivation, which increases the cost of planting. Although the total amount of phosphorus (P) in the soil may be high, plants mainly absorb P in the inorganic form (Pi), which is present at a low concentration, limiting plant growth and development [2].

Phosphorus is an essential macronutrient for all living organisms and plays important roles in energy metabolism; biosynthesis of nucleic acids, phospholipids and membranes; cellular signal transduction and the regulation of many enzymes [3,4]. Plants have evolved two broad strategies to cope with phosphate starvation, which involve changes in physiology, biochemistry and root morphology that enhance their ability to activate, assimilate and transport insoluble phosphate in soils [5].

Low-phosphate stress not only increases root biomass but can also cause significant changes in root morphology, including altering the root-to-shoot ratio, total root length and lateral root length and numbers, to increase the contact area with the soil; these changes improve the absorptive capacity of roots. Low phosphate availability has been found to favor lateral root growth over primary root growth by dramatically reducing primary root length and increasing lateral root elongation and lateral root density in *Arabidopsis*[5,6]. In white lupine, the rate of proteoid root formation is found to be greatest in solutions with a Pi concentration of 1–10 mmol m^-3^ and is suppressed at concentrations of 25 mmol m^-3^ Pi and higher [7]. The effect of low phosphate levels on the rice root system is found to be induction of adventitious root growth and increases in the number and length of lateral roots, which increase the surface area available for nutrient uptake by roots [8]. The root-to-shoot ratio has been observed to increase in maize when plants were subjected to Pi deficiency, although the reported effects on root length and biomass are inconsistent. Anghinoni and Barber [9] reported that root length was increased in 12-day-old seedlings when they were cultured under low-phosphate conditions. In contrast, Mollier and Pellerin [10] observed that the elongation rate of axile roots was maintained throughout the experimental period, whereas the emergence of new axile roots and the elongation of first-order lateral roots were dramatically reduced. These differences may be due to differences in the experimental conditions and genotypes involved, and they suggest that there is a high degree of plasticity in maize roots.

In recent years, several components involved in Pi starvation signaling in plants have been identified and characterized in detail. PHR1, a conserved MYB transcription factor, is shown to regulate microRNA399 (miR399) family members and a number of other Pi starvation response genes [11,12]. Recently, the *OsPHR1* and *OsPHR2* genes of rice have been cloned, both of which are involved in the Pi starvation response. Overexpression of *OsPHR2* induces a Pi starvation response, suggesting that this gene plays a conserved upstream role in Pi signaling regulation [13]. Three additional transcription factors, OsPTF1/ZmPTF1, WRKY75 and ZAT6 (zinc finger of *Arabidopsis* 6, a cysteine-2/histidine-2 zinc finger protein), are found to participate in the regulation of plant adaptation to phosphate starvation [14-17]. Moreover, miR399 has been found to be involved in the posttranscriptional regulation of Pi homeostasis [12,18-20]. Recently, Pant *et al.*[20] reported that miR399 is a long-distance signal for the regulation of Pi homeostasis in the phloem. Several Pi phosphate transporters and key enzymes that produce organic acids have been identified, the upregulation of which could increase the uptake and use efficiency of Pi within a plant [21]. Although certain components of Pi starvation signaling in plants have been identified, the overall pathway is still poorly understood and requires further investigation.

High-throughput macro/microarray technologies have contributed enormously to demonstrating the transcriptional regulation associated with abiotic stresses, including low levels of inorganic Pi. Hammond *et al.* identified *Arabidopsis* genes for which the expression in the leaves increases specifically in response to P starvation when the P content in plant tissues begins to decline, but before the lack of P affects growth, and these researchers identified marker genes to monitor P deficiency in plants [22]. Based on the expression analysis of plants during a 3-d period after the removal of Pi from the growth medium, Wu *et al.* suggested that a significant fraction of regulatory genes exhibit distinct or even contrasting expression patterns in the leaves and roots of *Arabidopsis* plants in response to Pi starvation, supporting the idea that distinct strategies are used in different plant organs in response to a shortage of Pi in growth media [23]. This hypothesis was confirmed by Misson *et al.* using Affymetrix gene chips [24]. In rice, Wasaki *et al.* found that sulfoquinovosyl diacylglycerol (SQDG) synthesis-related genes and polysaccharide metabolism were affected by Pi levels [25,26]. Calderon-Vazquez *et al.* examined transcript profiles of *Zea mays* roots and revealed gene responses to phosphate deficiency at the plant- and species-specific levels [27]. Gene expression analyses of responses to phosphorus deficiency are also performed in proteoid roots of white lupin [28] and roots of the common bean [29]. A large number of differentially expressed genes have been discovered using macro/microarrays. A proteomics approach was used to identify proteins that are differentially expressed under low-phosphate conditions and among different inbred lines [30,31]. Taken together, these findings suggest four main changes when plants are subjected to low-phosphate conditions: 1) phosphorus absorption and utilization-related genes, such as phosphate transporters, acid phosphatases, organic acid synthases and nucleases, which could improve Pi absorption and release Pi from internal and external environments, are induced when plants are subjected to low-phosphate conditions; 2) lipid metabolism and membrane components are altered by the substitution of P with sulfur in various types of lipids; 3) primary modes of metabolism, such as carbon metabolism and nitrogen metabolism, are affected by a lack of phosphate; and 4) there are changes in gene expression related to the response to metallic elements and other abiotic stresses. The results of high-throughput analysis give us a better understanding of plant responses to phosphate starvation, but little is known regarding the plant root modifications that occur under low-phosphate conditions and their regulatory mechanisms. The available evidence suggests that auxin plays an important role in mediating the effects of Pi starvation on root system architecture. Phosphate availability alters lateral root development in *Arabidopsis* at least partly by modulating auxin sensitivity via a mechanism involving the TIR1 auxin receptor [32,33].

In this study, the response to phosphate starvation of the roots of maize plants from the inbred line Q319 was analyzed. The numbers of lateral roots and lateral root primordia decreased after 6 days of culture in a low-phosphate solution (LP) compared with those of plants grown under normal conditions (sufficient phosphate, SP), and these differences increased in association with the stress caused by phosphate starvation. However, the growth of primary roots appeared not to be sensitive to low phosphate levels. This finding differed from what is observed in *Arabidopsis*; when the root tip of an *Arabidopsis* plant comes into contact with low-phosphate media, primary root growth ceases. To elucidate how low phosphate levels regulate root modifications, especially lateral root development, a transcriptomic analysis of the 1.0-1.5 cm lateral root primordium zone (LRZ) of maize Q319 roots was completed. The data analysis showed that auxin signaling participated in the response to low-phosphate conditions and the modification of root morphology, and LOB (Lateral organ boundaries) domain proteins might represent an intermediary between auxin signaling and root morphology. The retardation of lateral root development may be caused by the coordinated downregulation of the genes involved in DNA replication, gene expression, protein synthesis and degradation and cell growth. These findings enrich our understanding of plant responses to low-phosphate conditions and maize root morphogenesis.

## Results

### Low-phosphate treatment retards shoot growth and promotes root growth in maize plants

**Figure 1:**
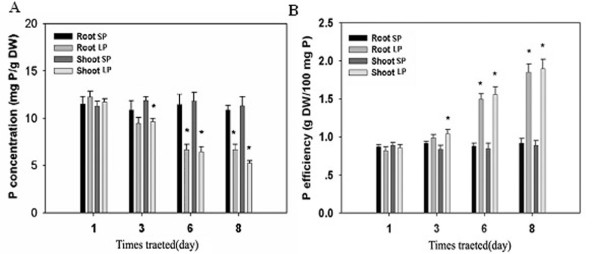
**Phosphate concentration (A) and phosphate efficiency (B) in Q319 maize seedlings in different nutrient solutions. Values are means ± sd**. The asterisks indicate significant differences between seedlings in the SP and LP nutrient solutions at the *0.05 level using a *t*-test (n = 8).

**Figure 2:**
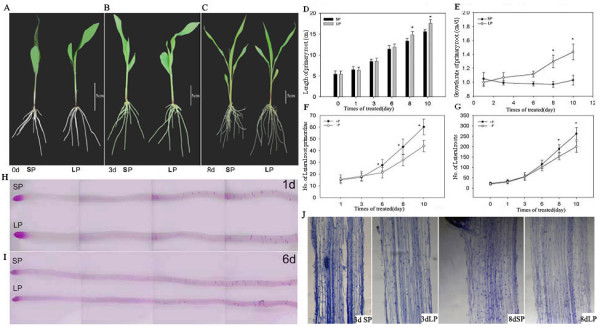
**Phenotype characteristics of maize plants of the Q319 line cultured in SP and LP nutrient solutions.** Q319 maize seedlings cultured in the SP or LP solution at different time points: (A) 0 d, (B) 3 d and (C) 8 days, and the length (D) and growth rate (E) of maize primary roots in the SP and LP nutrient solutions. Numbers of lateral root primordia (F) and lateral roots (G); lateral root primordia subjected to Feulgen staining (H, I); and paraffin sections of the LRZ of roots (J) in the SP and LP solutions.

As shown in Table 1 and Figure 1, after 3 days of LP treatment, the Pi contents in both roots and shoots had decreased slightly compared with those of plants cultured in the SP solution, whereas the growth and morphology of these plants did not show significant differences. In conjunction with the stress caused by phosphate starvation, the difference in the Pi content between plants under LP and SP conditions was increased, and the root/shoot P content ratios under LP conditions were also higher compared with SP conditions. The Pi concentrations in both roots and shoots were reduced after 3 days of culture in the LP solution compared with those in the plants in the SP solution, and the difference in the Pi concentrations in the shoots was significant at the 0.05 level based on a *t*-test (Figure 1A). This finding was consistent with results obtained in *Arabidopsis*. After 6 days in the LP solution, the Pi concentrations in both shoots and roots were approximately half of those of the plants in the SP solution, and the Pi efficiency was approximately 167 % of that in the SP solution (Figure 1). The dry weight of the roots from the plants in the LP solution increased after 3 days of treatment compared with the SP condition. Figure 2 and Table 2 show that the growth of shoots was clearly suppressed after 8 days of culture in the LP nutrient solution, whereas root growth was promoted, especially that of primary roots. After 8 days of culture, the dry weight of the roots in the LP solution was 1.16-fold higher than the weight of roots in the SP solution, and this difference reached a factor of 1.20 after 10 days of culture. In contrast, the dry weight of the shoots of the plants in the LP solution was lower than the weight observed under SP conditions. In the growth of the plants, the R/L ratio was reduced in both the LP and SP solutions, but the ratios of the plants in the LP solution were higher than those observed for SP plants (Table 2). When the P content and R/L ratio were compared, we found that the plants allocated more phosphorus to their roots to ensure root growth when they were subjected to low-Pi conditions, and the well-developed roots provided a greater area of contact with the medium, which was beneficial for Pi uptake.

**Table 1:** Phosphorus contents in the root and shoot of Q319 in nutrient solutions

**Time (day)**	**P content in Root (mg P/plant)**		**P content in Shoot (mg P/plant)**		**P content in Root/P content in Shoot**	
	SP	LP	SP	LP	SP	LP
1	15.74 ± 1.57	16.98 ± 1.70	30.38 ± 3.15	31.59 ± 2.61	0.52 ± 0.01	0.53 ± 0.02
3	19.01 ± 1.78	17.03 ± 2.04	41.22 ± 3.02	33.87 ± 2.65*	0.46 ± 0.02	0.50 ± 0.02
6	28.10 ± 2.78	17.76 ± 1.77*	69.56 ± 5.88	38.77 ± 3.62*	0.40 ± 0.01	0.46 ± 0.02*
8	30.39 ± 2.84	17.47 ± 1.93*	95.63 ± 7.27	37.63 ± 4.49*	0.32 ± 0.01	0.45 ± 0.03*
10	28.45 ± 3.04	14.53 ± 1.45*	103.9 ± 7.91	33.95 ± 3.96*	0.27 ± 0.03	0.43 ± 0.02*

Values are the means ± sd. The asterisks indicate significant differences between seedlings in SP and LP solutions at the *0.05 level using the *t*-test (n = 8).

**Table 2:** The biomass of maize Q319 in SP and LP solutions

**Time (day)**	**Root DW (10^-2^ g/plant)**	**Shoot DW (10^-2^ g/plant)**	**Ratio of root-shoot**			
	SP	LP	SP	LP	SP	LP
0	1.20 ± 0.11	1.17 ± 0.09	2.34 ± 0.12	2.29 ± 0.10	0.51 ± 0.02	0.51 ± 0.01
1	1.37 ± 0.065	1.39 ± 0.17	2.70 ± 0.04	2.70 ± 0.13	0.50 ± 0.02	0.51 ± 0.02
3	1.75 ± 0.21	1.80 ± 0.20	3.47 ± 0.48	3.51 ± 0.49	0.50 ± 0.03	0.54 ± 0.04
6	2.46 ± 0.17	2.67 ± 0.20	5.88 ± 0.47	6.03 ± 0.39	0.42 ± 0.02	0.44 ± 0.01
8	2.79 ± 0.20	3.23 ± 0.18*	8.47 ± 0.30	8.09 ± 0.36	0.33 ± 0.03	0.40 ± 0.03
10	3.04 ± 0.14	3.66 ± 0.22*	10.57 ± 0.23	9.51 ± 0.27*	0.29 ± 0.01	0.37 ± 0.03*

Values are the means ± sd. The asterisks indicate significant differences between seedlings in SP and LP nutrient solutions at the *0.05 level using the *t*-test (n = 10).

### Modification of the root system of maize subjected to LP stress

The growth of primary roots and lateral roots was investigated under various Pi supply levels. After 8 days of culture, there was a distinct difference in the root system architecture between the plants in the SP and LP solutions (Figure 2A-C). After 6 days of treatment, the primary roots of the plants in the LP solution were longer than those of the plants in the SP solution, and after 8 days of treatment, the difference was significant at the 0.05 level based on a *t*-test. The growth rate of primary roots in the LP solution was 1.5-fold higher than that of the plants in the SP solution after 10 days (Figure 2D-E). The cell morphology of the elongation zone in maize roots was analyzed by staining the paraffin sections with hematoxylin, and the cell lengths did not differ considerably between the two treatment conditions (Figure 2 J). The numbers of lateral roots and lateral root primordia were reduced after 6 days of treatment in the LP solution, and this difference increased in association with the stress caused by phosphate starvation (Figure 2 F-I). It was concluded that when maize plants were cultured in the LP solution, the growth of primary roots was promoted, whereas the formation of lateral roots was inhibited.

### Microarray analysis of the lateral root primordium zone (LRZ)

**Figure 3:**
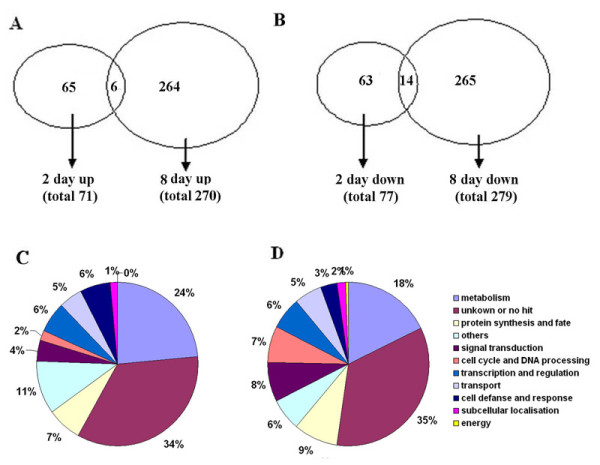
Venn diagram illustrating the number of differentially expressed genes that are up- (A) or downregulated (B) in response to low phosphate in either or both treatments, 2 days or 8 days, in the maize LRZ and functional classification of genes that are differentially expressed in the maize LRZ following 2 days (C) and 8 days (D) of low phosphate treatment.

The microarray used in this study contained 46,000 70-mer oligonucleotides for maize genes, representing >30,000 unique identifiable maize genes (details at http://www.maizearray.org). The alterations of the global genome associated with the response of the LRZ region to low phosphate (2 days and 8 days) were determined. The LRZ region was defined as the 1.0-1.5 cm segment containing the lateral root primordia. Transcripts exhibiting a difference between treated and control plants of more than 1.5-fold or less than 0.66-fold and a p value of less than 0.05 determined by a *t*-test were identified as differentially expressed transcripts for the purpose of searching for genes induced or inhibited by phosphate. After 2 days of treatment, 148 transcripts were found to be differentially expressed, with 71 transcripts upregulated and 77 downregulated. After 8 days of low-phosphate treatment, 549 genes were differentially expressed, with 270 genes upregulated and 279 downregulated (Additional files 1). As shown in Figure 3, a total of 20 genes showed similar trends at the two time points. These differentially expressed genes were divided into several functional categories according to biological process annotation. In the LRZ, after treatment for 2 days, the largest functional group of these genes was associated with metabolism (24 %); the second largest was the group of other genes (11 %) that could not be classified into any major functional category; and the third largest were the groups of genes involved in cell defense and response (6 %) and transcription and regulation (6 %) (Figure 3 C). After 8 days of low-phosphate treatment, the largest functional groups of genes differentially expressed in the LRZ were related to metabolism (18 %), protein synthesis and fate (9 %), signaling transduction (8 %) and cell proliferation and regulation (7 %), with the exception of the genes of unknown function (Figure 3D). These results revealed that the numbers and functional categories of genes that were differentially expressed in the LRZ were modified by the severity of the low-phosphate treatment. The data have been deposited into the GEO database under Accession No. GSE36368.

### Validation of expression analysis by real-time RT-PCR (qPCR)

To confirm the results in the microarray analysis, 29 genes with different transcript abundances were validated by real-time RT-PCR. Considering the sensitivity of the microarray, the expression of these genes showed good consistency between the two detection methods. For example, the *SPX* and *PTF* genes were upregulated, as has been reported in other plants. The primers used in this analysis and its results are listed in Additional files 2 and Additional file 3.

### Auxin participate in the regulation of lateral root development under LP conditions

**Figure 4:**
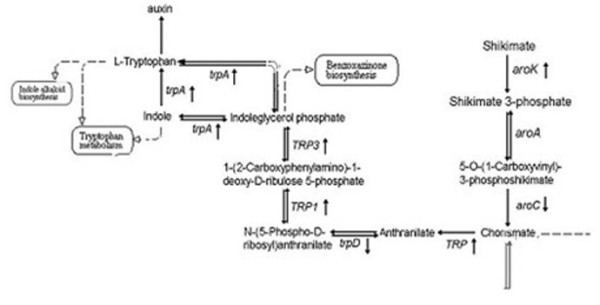
**Modulation of genes encoding enzymes involved in auxin metabolism in response to low phosphate stress.** Differentially modulated genes are indicated with an arrowhead. Biochemical and physiological pathways were classified according to the KEGG database (http://www.genome.jp/kegg/). aroK: shikimate kinase; aroA: 3-phosphoshikimate 1-carboxyvinyltransferase; aroC: chorismate synthase; TRP: anthranilate synthase/indole-3-glycerol phosphate synthase; trpD: anthranilate phosphoribosyltransferase; TRP1: anthranilate synthase/indole-3-glycerol phosphate synthase/phosphoribosylanthranilate isomerase; TRP3: anthranilate synthase/indole-3-glycerol phosphate synthase; trpA: tryptophan synthase alpha chain.

Phytohormones play important roles in root morphogenesis. Table 3 lists differentially expressed genes that are related to phytohormones. These genes are involved in the metabolism, detection and signal transduction of auxin, ethylene, gibberellin, ABA and cytokinins. Interestingly, the synthesis of auxin in the LRZ appears to be differentially regulated by phosphate levels. Figure 4 shows the main steps involved in IAA synthesis and the changes in the expression levels of several genes involved in this pathway under LP conditions. Transcripts corresponding to enzymes that play a role in the synthesis of tryptophan were the most upregulated. For example, *shikimate kinase* (*arK*, TM00030640, TM00015709) were 1.81- and 2.22-fold upregulated. The *tryptophan synthase alpha chain* (*trpA,* TM00027996) was upregulated 1.94-fold and the *anthranilate synthase alpha 2 subunit* (TM00014943) was 1.64-fold upregulated compared with plants in SP solution. Moreover, *anthranilate synthase/indole-3-glycerol phosphate synthase* (*TRP*), which encodes a product that catalyzes the irreversible conversion of chorismate to anthranilate, was also upregulated by real-time RT-PCR assay (Additional file 4). These differentially expressed genes play key roles in the Trp-dependent IAA biosynthetic pathway. Auxin influx/efflux transporters, which are involved in the polar transport of auxin, were also affected by low-phosphate treatment. The *AUX1 protein* (TM00015932) was downregulated under LP conditions. The expression of *LOB domain protein 17* (TM00030748), which is similar to LOB domain protein LBD29 in *Arabidopsis*, increased after both 2 days and 8 days, by 1.74-fold and 4.21-fold, respectively. LOB domain protein is a key regulator of post-embryonic root initiation that is regulated by auxin [34,35] and may represent an intermediary between auxin signaling and root morphology. In summary, the synthesis and transport of auxin in the maize LRZ were altered when plants were subjected to low-phosphate conditions. The differential expression of these genes involved in regulating auxin concentrations and signaling may contribute to the differences in lateral root development observed under SP and LP conditions, such as the inhibition of lateral root development and the promotion of primary roots.

**Table 3:** Genes expressed differentially related to plant hormone and other signaling

**Tigr_ID**	**Putative_Annotation**	**Ratios of transcript abundance**	**(LP treated/SP control)**
	Auxin	2 day	8 day
			
TM00024179	3-phosphoshikimate 1-carboxyvinyltransferase {Zea mays;}	0.93	0.63^*^
TM00027996	putative tryptophan synthase alpha chain {Oryza sativa (japonica cultivar-group);}	0.79	**1.94**
TM00014943	anthranilate synthase alpha 2 subunit {Oryza sativa (japonica cultivar-group);}	0.94	**1.64**
TM00043903	putative anthranilate phosphoribosyltransferase {Oryza sativa (japonica cultivar-group);}	**1.62**	0.88
TM00030640	shikimate kinase {Oryza sativa (japonica cultivar-group);}	1.22	**1.81**
TM00015709	shikimate kinase {Oryza sativa (japonica cultivar-group);}	1.17	**2.02**
TM00015932	AUX1 protein {Zea mays;}	0.77	**0.54**
TM00030748	putative LOB domain protein 17 {Oryza sativa (japonica cultivar-group);}	**1.98**	**4.21**
			
TM00026596	ethylene responsive element binding factor3 {Oryza sativa (japonica cultivar-group);}	0.68	**0.52**
TM00016033	putative ethylene-responsive element binding factor {Oryza sativa (japonica cultivar-group);}	0.88	**0.44**
			
TM00033475	beta-D-glucosidase (EC 3.2.1.-) glu2 precursor - maize {Zea mays;}	**2.06**	0.67
TM00036348	response regulator 4 {Zea mays;}	**1.68**	1.03
			
TM00025193	gibberellin-induced receptor-like kinase TMK {Oryza sativa (japonica cultivar-group);}	1.03	**0.6**
TM00043843	putative ZmGR1a {Oryza sativa (japonica cultivar-group);}	**0.57**	**0.57**
			
TM00028288	putative abscisic acid-induced protein {Oryza sativa (japonica cultivar-group);}	**1.88**	**1.54**
			
TM00031932	putative Rop family GTPase ROP5 {Zea mays;}	-	**0.39**
TM00017643	putative Rop family GTPase ROP8 {Zea mays;}	**0.58**	1.07

^*^ Values means that the ratio of signal intense (LP treated/SPcontrol), and bold means that it meet the criterion of differentially expressed transcripts.

### Differentially expressed genes in ethylene, cytokinins and gibberellin and rop signaling pathway

In the maize LRZ, a β-glucosidase (TM00033475) that converts cytokinins from an inactive to an active form was upregulated 2.06-fold by low-phosphate conditions. The level of *β*-glucosidase protein was found to be reduced in the whole root based on a proteomic analysis performed by Li *et al.*[30]. Homolog gene of *ARABIDOPSIS RESPONSE REGULATOR 4* (*ARR4,*TM00036348) was found to be differentially expressed in the LRZ on day 8 under LP conditions (Table 3). The response of the lateral roots and primary roots of maize might be extended to other tissues or for a longer period due to signaling regulated by different ratio of auxin to cytokinins or different cytokine concentrations.

Ethylene may also play a role in lateral root development because auxins are thought to trigger ethylene production in roots [36]. Two ethylene responsive element binding factors (TM00026596 and TM00016033) were 0.44- and 0.52-fold downregulated by low-phosphate conditions. Additionally, two gibberellin-responsive genes (TM00025193 and TM00043843) and a putative abscisic acid-induced protein (TM00028288) were also differentially expressed between the LP and SP conditions (Table 3). The Rop GTPases are a plant-specific subfamily of small G proteins that participate in various processes in plants [37]. There are 9 Rop GTPases in maize. In the LRZ of plants treated with the low-phosphate solution, the Rop family *GTPases, Rop5* (TM00031932) and *Rop8* (TM00017643), were downregulated (Table 3). The described above suggested that various signaling pathways might be involved in the acclimation of plants to low-phosphate and lateral root development.

### Transcription factors and cell cycle-related genes contribute to lateral root emergence

Table 4 lists the transcription factors involved in the response to low phosphate. A total of 27 transcription factors, including zinc-finger proteins (C3HC4-type RING finger and C2H2-type RING finger), a b-helix-loop-helix domain protein, MYB transcription factors, bZIP transcription factors, AP2/DREBP domain-containing proteins, NAC domain proteins and other putative transcription factors, were differentially expressed in the LRZ between the plants in the LP and SP nutrient solutions at one or more time points during the culture. Among this group, the b-helix-loop-helix domain protein PTF1 and MYB transcription factors were upregulated in the response to low phosphate.

**Table 4:** Differentially expressed transcription factors

**Tigr_ID**	**Putative_Annotation**	**Ratios of transcript abundance**	**(LP treated/SP control)**
		2 day	8 day
TM00015861	AP2 transcriptional activator DRF1.3 {Hordeum vulgare;}	0.89	**1.77**
TM00025480	putative DREPP2 protein {Oryza sativa (japonica cultivar-group);}	0.45	**0.35**
TM00018876	bZIP transcription factor {Oryza sativa (japonica cultivar-group);}	**2.02**	0.95
TM00016473	C2 domain-containing protein-like {Oryza sativa (japonica cultivar-group);}	1.21	**1.96**
TM00017384	C2 domain-containing protein-like {Oryza sativa (japonica cultivar-group);}	1.34	**1.81**
TM00019240	C2H2 type zinc finger containing protein {Oryza sativa (japonica cultivar-group);}	1.43	**2.32**
TM00016681	C2H2 zinc-finger protein {Zea mays;}	**2.02**	1.42
TM00041812	RING zinc finger protein-like {Oryza sativa (japonica cultivar-group);}	**1.54**	0.7
TM00016816	CHP-rich zinc finger protein-like {Oryza sativa (japonica cultivar-group);}	0.82	**0.62**
TM00029057	FIERG2 protein - rice {Oryza sativa;}	1.35	**2.6**
TM00020377	putative Dof zinc finger protein {Oryza sativa (japonica cultivar-group);}	0.83	**0.63**
TM00057274	zinc finger protein {Oryza sativa (indica cultivar-group);}	1.1	**1.57**
TM00041802	zinc finger protein {Oryza sativa (japonica cultivar-group);}	0.87	**1.54**
TM00033445	zinc finger protein {Oryza sativa (japonica cultivar-group);}	1.44	**0.62**
TM00020992	zinc finger transcription factor WRKY1 {Oryza sativa;}	**0.66**	1.21
TM00041105	zinc finger transcription factor ZFP30 {Oryza sativa (japonica cultivar-group);}	0.81	**0.52**
TM00000942	putative Myb-related transcription factor {Oryza sativa (japonica cultivar-group);}	1.25	**0.61**
TM00044596	R2R3MYB-domain protein {Zea mays;}	**1.59**	2.83
TM00001709	putative WRKY transcription factor {Oryza sativa (japonica cultivar-group);}	**1.59**	1.33
TM00040147	putative WD-40 repeat protein {Oryza sativa (japonica cultivar-group);}	**0.62**	0.75
TM00018559	putative WD-40 repeat protein {Oryza sativa (japonica cultivar-group);}	1.1	**1.73**
TM00013450	putative transcription factor {Oryza sativa (japonica cultivar-group);}	**0.96**	**0.63**
TM00017631	putative transcription factor {Oryza sativa (japonica cultivar-group);}	**0.93**	**0.44**
TM00014473	putative translation factor {Pinus pinaster;}	**1.51**	**3.14**
TM00031271	Transcription Factor {Oryza sativa;}	**1.22**	**4.45**
TM00030906	transcription factor Rap212 {Oryza sativa;}	**0.55**	**2.18**
TM00016712	transcription factor-like {Oryza sativa (japonica cultivar-group);}	**1.01**	**0.62**

^*^ Values means that the ratio of signal intense (LP treated/SPcontrol), and bold means that it meet the criterion of differentially expressed transcripts.

Several genes involved in the regulation of cell division, such as *cyclin Ia* (TM00041779), *proliferating cell nuclear antigen* (TM00041533), *cyclin-like protein* (TM00056744) were downregulated in the LRZ of plants in the LP culture for 8 days (Table 5), whereas While the *putative ripening-regulated protein DDTFR18* (TM00026516) was found to be upregulated after 2 days in the LRZ of the plants cultured in the LP solution. These results indicated that the cells in the LRZ were sensitive to LP culture conditions and began to exhibit decreased cell division. It could be inferred that these genes participated in the regulation of cell division and led to the reduction of cell division in the LRZ and decreased lateral root formation under LP conditions.

**Table 5:** Differentially expressed cell cycle related genes

**Tigr_ID**	**Putative_Annotation**	**Ratios of transcript abundance**	**(LP treated/SP control)**
		2 day	8 day
TM00041779	cyclin Ia - maize {Zea mays;}	0.95	**0.5**
TM00056744	cyclin-like protein {Oryza sativa (japonica cultivar-group);}	1.36	**0.31**
TM00041533	proliferating cell nuclear antigen {Zea mays;}	0.69	**0.38**
TM00042158	ripening-related protein-like {Oryza sativa (japonica cultivar-group);}	0.92	**0.64**
TM00024846	senescence-associated protein-like {Oryza sativa (japonica cultivar-group);}	-	**1.66**
TM00014046	Induced stolen tip protein TUB8 (Fragment). {Solanum tuberosum;}	0.95	**0.65**
TM00026516	putative ripening regulated protein DDTFR18 {Oryza sativa (japonica cultivar-group);}	**1.62**	1.56

^*^ Values means that the ratio of signal intense (LP treated/SPcontrol), and bold means that it meet the criterion of differentially expressed transcripts.

### Histone synthesis and nucleosome assembly were retarded under LP conditions

Histone genes showed an interesting expression pattern in the LRZ under LP conditions. Because histones are components of the nucleosome, their abundance may be an important signal of chromatin replication and cell proliferation. Almost all of the histone genes showed reduced expression in the LRZ under the LP conditions, but the rate of this reduction varied (Table 6). In the LRZ of plants cultured for 2 days in the LP solution, only a few genes encoding histone H3 were clearly downregulated. In the LRZ of plants cultured for 8 days under LP conditions, the expression levels of the histone H1, histone H2A, histone H2B, histone H3 and histone H4 coding genes were approximately half the levels under SP conditions. These findings indicated that nucleosome assembly and chromatin replication were retarded by the low-phosphate stress applied.

**Table 6:** Differentially expressed cell proliferation and growth related genes

**Tigr_ID**	**Putative_Annotation**	**Ratios of transcript abundance**	**Ratios of transcript abundance**
		2 day	8 day
	Histone		
TM00043106	histone H1 - maize {Zea mays;}	0.8	**0.48**
TM00034952	histone H1-like protein HON101 {Zea mays;}	0.72	**0.49**
TM00041038	histone H1-like protein {Zea mays;}	0.96	**0.49**
TM00057303	histone H1-like protein {Zea mays;}	0.95	**0.47**
TM00041037	histone H1-like protein {Zea mays;}	0.79	**0.36**
TM00043108	histone H1 - maize {Zea mays;}	0.8	**0.48**
TM00056330	histone H1 - maize {Zea mays;}	0.78	**0.36**
TM00034974	histone H2A {Oryza sativa (japonica cultivar-group);}	0.78	**0.44**
TM00013559	Histone H2A. {Cicer arietinum;}	0.84	**0.54**
TM00036839	Histone H2A. {Zea mays;}	0.78	**0.61**
TM00040901	Histone H2A. {Zea mays;}	0.76	**0.61**
TM00013381	Histone H2A. {Zea mays;}	0.7	**0.41**
TM00057312	Putative histone H2A {Oryza sativa (japonica cultivar-group);}	0.63	**0.53**
TM00013393	Histone H2B. {Triticum aestivum;}	0.89	**0.63**
TM00013518	Histone H2B.1. {Zea mays;}	0.94	**0.64**
TM00040846	Histone H2B.2. {Zea mays;}	0.8	**0.46**
TM00035010	Histone H2B.4. {Zea mays;}	0.88	**0.6**
TM00042818	histone H3 (clone pH3c-1) – alfalfa {Medicago sativa;}	0.79	**0.46**
TM00042822	histone H3 {Oryza sativa (japonica cultivar-group);}	**0.54**	**0.47**
TM00057261	histone H3. {Helicoverpa zea;}	**0.63**	**0.48**
TM00023730	histone H4 - Arabidopsis thaliana {Arabidopsis thaliana;}	0.75	**0.42**
TM00043088	histone H4 - Arabidopsis thaliana {Arabidopsis thaliana;}	0.7	**0.45**
TM00037330	histone H4. {Arabidopsis thaliana;}	0.91	**0.53**
TM00013350	histone H4. {Arabidopsis thaliana;}	0.82	**0.49**
	Ribosomal protein		
TM00013705	60 S ribosomal protein L2 (L8) (Ribosomal protein TL2). {Lycopersicon esculentum;}	**0.58**	0.4
TM00035320	putative 60 S ribosomal protein L27a {Oryza sativa (japonica cultivar-group);}	0.68	**0.62**
TM00023944	putative 60 S ribosomal protein L30 {Oryza sativa (japonica cultivar-group);}	**0.62**	0.65
TM00023903	putative 60 S ribosomal protein L37 {Oryza sativa (japonica cultivar-group);}	**0.64**	0.99
TM00024198	putative 40 S ribosomal protein S8 {Zea mays;}	1.38	**2.12**
TM00040764	putative ribosomal protein S17 {Oryza sativa (japonica cultivar-group);}	1.3	**0.61**
TM00056490	ribosomal protein L30p family-like {Oryza sativa (japonica cultivar-group);}	1.09	**0.6**
TM00014222	ribosomal protein s6 RPS6-2 {Zea mays;}	0.83	**0.56**
TM00027666	putative ribosomal protein {Oryza sativa (japonica cultivar-group);}	0.87	**0.54**
TM00034882	Chloroplast 30 S ribosomal protein S7. {Zea mays;}	1.33	**1.93**
	Protein synthesis		
TM00042967	elongation factor {Saccharum officinarum;}	0.96	**0.62**
TM00039650	elongation factor 1 alpha {Saccharum hybrid cultivar CP65-357;}	0.81	**0.56**
TM00037693	elongation factor 1 alpha {Zea mays;}	**0.53**	**0.42**
TM00014515	Eukaryotic initiation factor 4A (eIF4A) (eIF-4A).	0.89	**0.65**
TM00056705	Eukaryotic translation initiation factor 3 subunit 10 (eIF-3 theta)	**0.6**	**0.76**
TM00043994	putative serine protease {Oryza sativa (japonica cultivar-group);}	0.67	**0.54**
TM00026276	At1g64230/F22C12_17 {Arabidopsis thaliana;}	1.14	**1.95**
	Protein degradation		
TM00043210	putative ubiquinol-cytochrome C reductase complex ubiquinone-binding protein {Oryza sativa (japonica cultivar-group);}	1.11	**1.56**
TM00024337	putative ubiquinone/menaquinone biosynthesis methyltransferase {Arabidopsis thaliana;}	1.15	**1.82**
TM00043415	putative ubiquinone/menaquinone biosynthesis methyltransferase {Arabidopsis thaliana;}	1.36	**1.99**
TM00042923	polyubiquitin - maize {Zea mays;}	**1.56**	0.91
TM00027604	Probable U3 small nucleolar RNA-associated protein 11 {Oryza sativa;}	1.3	**2.21**
	Cell growth and expansion		
TM00024972	beta-expansin 7 {Zea mays;}	**0.56**	**0.42**
TM00024970	beta-expansin 7 {Zea mays;}	0.74	**0.40**
TM00033434	putative cellulase {Oryza sativa (japonica cultivar-group);}	**-**	**0.41**
TM00020258	cellulose synthase catalytic subunit 10 {Zea mays;}	1.38	**0.39**
TM00037072	cellulose synthase catalytic subunit 11 {Zea mays;}	1.38	**0.5**

^*^ Values means that the ratio of signal intense (LP treated/SPcontrol), and bold means that it meet the criterion of differentially expressed transcripts.

**Table 7:** Differentially expressed cell defense and response genes

**Tigr_ID**	**Putative_Annotation**	**Ratios of transcript abundance**	**(LP treated/SP control)**
		2 day	8 day
TM00026730	trehalose-6-phosphate phosphatase {Oryza sativa (japonica cultivar-group);}		**1.74**
TM00033100	putative trehalose-6-phosphate phosphatase {Arabidopsis thaliana;}	**1.88**	-
TM00030089	putative protein phosphatase-2 C {Oryza sativa (japonica cultivar-group);}	0.98	**0.32**
TM00024044	putative inorganic pyrophosphatase {Oryza sativa (japonica cultivar-group);}	0.98	**2.02**
TM00043868	probable nucleoside triphosphatase [imported] - Arabidopsis thaliana {Arabidopsis thaliana;}	1	**1.57**
TM00021634	putative 3(2),5-bisphosphate nucleotidase {Oryza sativa (japonica cultivar-group);}	1.18	**1.66**
TM00025464	RNaseP-associated protein-like {Oryza sativa (japonica cultivar-group);}	0.91	**1.53**
TM00004129	SPX domain containing protein {Oryza sativa (japonica cultivar-group);}	**2.49**	**4.38**
TM00020084	transporter-like protein {Arabidopsis thaliana;}	**1.85**	0.9

^*^ Values means that the ratio of signal intense (LP treated/SPcontrol), and bold means that it meet the criterion of differentially expressed transcripts.

### Protein synthesis and degradation and cell growth-related genes are affected by low-phosphate stress

Several genes encoding subunits of ribosome proteins were downregulated by low phosphate, but three ribosomal protein-coding genes (TM00024198 and TM00034882) were upregulated (Table 6). Ribosomes are the workhorses of protein biosynthesis, and their density determines the activity of protein synthesis in cells. The reduced expression of genes encoding subunits of ribosome proteins could restrict protein synthesis and retard lateral root development. Factors involved in protein synthesis, such as *translation initiation factor 3 subunit 10* (TM00056705), *initiation factor 4A* (*eIF4A*) (TM00014515), *elongation factor 1 alpha* (TM00037693, TM00039650) and *elongation factor* (TM00042967), were also downregulated in the LRZ under LP conditions. Based on these results, we were able to conclude that LP culture conditions decreased protein synthesis, and it is possible that a mechanism involved in the regulation of protein synthesis might play a role in the response to the LP stress.

Protein degradation was clearly accelerated in the LRZ under LP conditions. After culture in the LP solution for 2 days, it was observed that *polyubiquitin* (TM00042923) was upregulated. In the LP solution after 8 days, genes including a *putative ubiquinone/menaquinone biosynthesis methyltransferase* (TM00043415), *putative ubiquinol-cytochrome C reductase complex ubiquinone-binding protein* (TM00043210), *putative ubiquinone/menaquinone biosynthesis methyltransferase* (TM00024337, TM00043415) and *probable U3 small nucleolar RNA-associated protein 11* (TM00027604) were upregulated (Table 6). The increased degradation of proteins in the LRZ indicated an adaptation of the plants to low-phosphate conditions, which suggested that the cells in the LRZ had experienced great changes in their structure and protein content. It could be inferred that these changes may be advantageous for enhancing the growth of primary roots by recycling phosphorus in the LRZ and mature zone under low-phosphate conditions.

After culture for 2 days in the LP solution, the *beta-expansin 7* (MZ00024972) gene was downregulated. Furthermore, after 8 days under LP conditions, the *beta-expansin 7* (TM00024970 and TM00024972) and *cellulose synthase* (TM00020258 and TM00037072) genes were all downregulated. Expansin, which is located in the cell wall, is a key factor in the expansion and elongation of cells, whereas cellulose synthase is a key enzyme involved in cell wall synthesis. These results suggest that the cell wall in the LRZ would reconfigure when subjected to low-phosphate solution.

### The expression of transporters and other genes upregulated under LP conditions

Phosphatases, nucleotidases and inorganic pyrophosphatase can release phosphate from phosphorus compounds. Genes encoding phosphatases were upregulated under low phosphate conditions (Table7). *Inorganic pyrophosphatase* (TM00024044) was observed to be upregulated in the LRZ after 8 days in the LP solution, which could improve the phosphorus use efficiency in cells. *Nucleotidases* (TM00021634 and TM00043868), which improve the utilization of organic phosphorus from nucleosides under low-phosphate conditions, were induced 1.57- to 1.66-fold (Table7). The *SPX* (*SYG1/Pho81/XPR1*) *domain gene* TM00004129 was also induced by low-phosphate conditions. Furthermore, expression of the *AtSPX1* and *AtSPX3* genes was induced by Pi starvation [38,39], and these genes have been proposed to play different roles in the Pi signaling network in *Arabidopsis*. It could be concluded that phosphorus efficiency was enhanced in LRZ cells under LP conditions.

## Discussion

### Under LP conditions, the growth of maize primary roots was promoted, and lateral root development was retarded

Figure 2C shows plants that were cultured for 8 days in the LP and SP solutions. The root systems of the plants in the LP solution were more developed than those of the plants in the SP solution. The length and growth rates of the primary roots were increased in the LP solution (Figure 2D-E), whereas the numbers of lateral roots and lateral root primordia were reduced (Figure 2 F-G). Regulation of root architecture led to a robust root system for exploring for phosphate in deeper soils. To understand whether the accelerated growth of the primary roots was the result of increases in cell length or in the number of cell divisions, the cell morphology of the elongation zone was analyzed by staining paraffin sections with hematoxylin (Figure 2 J). The cell length did not present clear differences between the plants under the two treatment conditions, although there were a slightly greater number of cells in the roots of LP plants. After 6 days of culture in the LP solution, the growth rate of primary roots was increased, whereas the number of lateral root primordia was significantly lower compared with those of the plants in the SP solution. This result suggested that the root tip region sensed the low phosphate concentration, resulting in a response leading to modification of the root architecture. The modifications observed in the root systems of maize plants subjected to the LP nutrient solution were different compared with what is observed in the model plant species *Arabidopsis.* In the latter species, the growth of primary roots ceased abruptly, whereas the elongation and density of lateral roots were increased when plants were subjected to low-phosphate conditions [5,6]. In maize, the growth of primary roots was promoted, and the formation of lateral roots was reduced. In *Arabidopsis*, the root volume is increased by enhancing the growth of lateral roots, whereas for maize, this volume increase mainly occurs by promoting the elongation of axial roots in a low-phosphate environment. The differences between the responses of *Arabidopsis* and *Zea mays* to low-phosphorus stress indicate that these species exhibit different mechanisms for regulating root morphogenesis under LP conditions.

### The distribution of phosphate and carbohydrate was altered by low-phosphate stress

Table 2 provides the dry weight, and Table 1 provides the phosphate contents of plants under SP and LP conditions. Associated with the stress caused by phosphate starvation, the dry weight of the shoots produced under LP conditions increased slowly compared with what was observed for the plants in the SP solution, and significant differences were detected between the plants in the SP and LP nutrient solutions after 10 days. The root dry weight increased more rapidly under LP conditions; after 6 days of culture in the LP solution, the dry weight of roots was increased by 1.08-fold compared with the SP condition, and this difference reached 1.20-fold after 10 days in the LP solution,and then reduced to 0.92-fold compared with the SP condition after 20 days in the LP solution in conjunction with more decreased shoot growth (0.68-fold) (unpublished data) for a long phosphate deficit. When the plants grew upward, the R/L ratio decreased, but the ratios for the plants in the LP solution were higher than in SP plants. Therefore, the growth of shoots was suppressed, and the development of roots was promoted by LP stress. Under LP conditions, the plants allocated a greater amount of phosphate to the roots than to the shoots, maintaining a relatively high phosphate level to satisfy root growth, whereas the growth of shoots was retarded. This result indicated that maize plant transport increased the amounts of carbohydrates to the roots to allow cell division and expansion to occur under LP conditions.

### Transcriptomic changes in the LRZ of maize plants were an important strategy for coping with phosphate starvation

The lateral root primordium zone of primary roots in maize consists of many types of cells, such as epidermis cells, cortex parenchyma, stele cells, lateral root primordium founders, and lateral root primordium cells, and is a region undergoing rapid growth and intricate cell differentiation. The density of the lateral root primordia in the LRZ was strongly influenced by the phosphate concentrations in the nutrient solutions. Each type of cell has unique transcriptomic properties. Therefore, the transcriptomic changes in the LRZ under LP conditions are expected to be extensive. In this study, we found that after culture for 2 days in the LP nutrient solution, 148 transcripts in LRZ were differentially expressed, with 71 being upregulated and 77 downregulated. Furthermore, after 8 days of low-phosphate treatment, 549 genes were differentially expressed, with 270 genes upregulated and 279 downregulated. Although the differential expression detected for certain genes represented the average of various types of cells in the lateral root primordium zone of primary roots, we found that under LP conditions, the three largest functional groups of repressed genes were involved in metabolism, protein synthesis and cell proliferation, with the exception of genes of unknown function, and that phytohormone signaling in the LRZ may play an important role in the response to low-phosphate culture conditions. This result suggests that the transcriptomic changes observed in the LRZ of maize plants are an important strategy to cope with phosphate starvation, rather than relying only on passive acclimation.

### Lateral root development under LP conditions was regulated by auxin biosynthesis in the LRZ

Certain genes related to phytohormone biosynthesis and signal transduction were differentially expressed in plants in the SP and LP solutions (Table 3). These genes are mainly involved in the synthesis of auxin metabolism and signal transduction. The effects of low-phosphate stress on root morphogenesis were caused, in part, by hormone signaling, and the phosphorus signal transduction pathway may engage in crosstalk with hormonal signaling pathways. Auxin biosynthesis increased in the LRZ under LP conditions, as transcripts corresponding to enzymes in the Trp-dependent IAA biosynthetic pathway were the most highly upregulated. With respect to the synthesis of tryptophan, *anthranilate synthase/indole-3-glycerol phosphate synthase* (*TRP*), a key enzyme that catalyzes the irreversible conversion of chorismate to anthranilate, was upregulated 3.09-fold compared with SP conditions (Additional file 4). Expression of LOB domain protein 17 (TM00030748) was increased at both 2 days and 8 days of culture in the LP nutrient solution. LOB domain protein is a key regulator of post-embryonic root initiation that is regulated by auxin [34,35] and that may be an intermediary between auxin signaling and root morphology. It was inferred that the phosphate supply influenced local auxin synthesis and its distribution, leading to modification of the root system in the LP solution. It is possible that a high level of auxin polar transport promotes primary root elongation and enhances auxin synthesis in the LRZ to ensure a basal auxin concentration for lateral root primordium formation in maize when subjected to low-phosphate conditions.

López-Bucio *et al.* reported that cytokinins inhibited lateral root formation in *Arabidopsis* plants under conditions of phosphate deficiency [40]. Martin *et al.* reported that increases in the ratio of root growth to shoot growth that occur in *Arabidopsis* in response to phosphate (Pi) deprivation were paralleled by a decrease in cytokinin levels under the same conditions and that exogenous cytokinins repressed the expression of *AtIPS1* and other Pi starvation-responsive genes [41]. *ARR4* (TM00036348) was upregulated in the LRZ of maize plants cultured in the LP solution for 2 days, following which its levels returned to the level observed in the plants in the SP solution. The type-A ARRs of the cytokinin two-component signaling system act as negative regulators of cytokinin signaling, with the exception of ARR4 [42]. The expression changes observed for *ARR4* under LP conditions suggested that the restriction of lateral root formation in maize under low-phosphate conditions could be an effect of cytokinin activity, and the ratio of cytokinins to auxin may play an important role in the modification of the root system under low-phosphate conditions. Also, GA and ethylene may be involved in the response to low-phosphate stress via crosstalk with other signaling pathways. Rops are a plant-specific subfamily of small G proteins that play a signaling role in diverse developmental processes and that regulate primary root elongation, lateral root formation, and root hair polarity in response to various environmental factors [43,44]. ROP5 and ROP8 showed down-regulated expression in the LRZ of maize to low-phosphate stress. It can be assumed that the promotion of the growth of primary roots and the retardation of the development of lateral root primordia under low-phosphate stress were associated with changes in Rop signaling.

### Transcription factors participate in the response to LP conditions in the LRZ

Zinc finger proteins (C3HC4-type RING finger and C2H2-type RING finger), bHLH proteins, MYB transcription factors, AP2/DREBP domain-containing proteins, NAC domain proteins and other putative transcription factor-encoding genes were differentially expressed in the LRZ under LP and SP conditions. Among these proteins, the b-helix-loop-helix domain protein *PTF1*, which was induced by phosphate starvation, has been found to play an important role in the development of roots. Overexpression of *PTF1* improves root development in rice and maize [14,15]. Formation of lateral root primordia is a key developmental event, and it could be inferred that *PTF1* regulates this process. The function of MYB transcription factors and the other differentially expressed transcription factors in the response to phosphorus starvation requires further study.

### Genes related to cell proliferation as well as protein synthesis and degradation showed differential expression under LP conditions

The growth rate of roots depends on cell division and cell elongation. Microarray analysis showed that genes related to nucleosome assembly and the cell cycle were differentially expressed in the LRZ under LP conditions. Almost all of the histone-coding genes showed reduced expression in the LRZ in the LP solution, suggesting that nucleosome assembly and chromatin replication in the LRZ were retarded or restricted under LP conditions.

The expression of genes encoding enzymes participating in cell growth and elongation, such as beta-expansin and cellulose synthase, decreased under LP conditions. Expansins, which localize to the cell wall, are a key factor in the expansion and elongation of cells, whereas cellulose synthase is a key enzyme involved in cell wall synthesis. These genes may directly function in the regulation of the lateral root and LRZ growth. Genes involved in protein synthesis, especially genes encoding subunits of ribosome proteins, were downregulated in the LRZ by low-phosphate stress, whereas protein degradation was accelerated. Ubiquitin and the 26 S proteasome participate in protein degradation, which may represent an adaptation mechanism in plants under LP conditions. These results suggested that the growth and division of cells in the LRZ were retarded by the phosphorus deficit and that the maize plants supported the growth of their primary roots by enhancing the recycling of phosphate in cells under LP conditions. Considering these results together with the fact that the primary roots showed accelerated growth under LP conditions, we were able to conclude that higher rates of cell growth and division occurred in the meristems of root tips subjected to the LP treatment compared with those of root tips under SP conditions.

### Gene expression patterns to LP conditions have tissue- specific and species-specific

Higher plants exhibit an amazing diversity and plasticity of root architectures. A large number of differentially expressed genes have been discovered using the whole root of *Arabidopsis* when subject to low phosphate conditions [23,24]. Pi starvation alters the balance of synthetic and catabolic carbon metabolisms, activates disease or pathogen resistance and toxin catabolism genes, causes protein synthesis down-regulated and the protein degradation up-regulated. MAP kinase cascades involved in the signal transduction cascades triggered by gibberellin, auxin and ethylene and certain transcription factors are participated in the response to low phosphate stress [23,24]. The cereal root system is more complex, and the branched roots are composed primarily of postembryonic adventitious roots. The expression analysis of rice and maize using whole root treated by low phosphate mainly focus on the influence to primary and secondary metabolism, Pi absorption and recycling and transcription regulators response to low phosphate [25,27]. Result showed that genes involved in metabolism and environment stress response were universal among these species. Certain factor related to root architecture has been found to be differentially expressed such as *ARF* and *AUX/IAA* TFs and *orthologues to SHORT-ROOT* and *SCR*[27]. The maize root system consists of different root types that are formed during different stages of development. The embryonic root system consists of a primary root that is formed at the basal pole of the embryo and a variable number of seminal roots that are laid down at the scutellar node, usually 3 or 4 seminal roots. And the postembryonic root system is composed of shoot borne roots that are formed at consecutive shoot nodes and lateral roots that are initiated in the pericycle of all roots [45]. Being a C4 plant, the cortical parenchyma consists of 10–15 cell layers, and the proximal root meristem is made up of hundreds of cells [45]. In this paper, the 1.0-1.5 cm LRZ of primary root was used to detect the gene expression involved in the root system acclimation to the low phosphate stress. The fewer number of differentially expressed genes may due to the less cell diversity of this segment. When subject to LP culture condition, certain genes involved in auxin biosynthesis and signaling, cell defense response protein degradation showed up-regulated, whereas the expression of genes participated in cell proliferation and growth were decreased. Several transcription factors showed differentially expressed under SP and LP conditions. Genes mainly functioned in meristem region and other tissues were not found to be upregulated or downregulated. These suggested that gene expression patterns to low phosphate conditions have tissue-specific and species-specific.

## Conclusions

In this study, we identified changes in root morphology when maize plants were cultured in an LP solution. The growth of primary roots was promoted, whereas the formation of lateral roots was inhibited. This result differed from what is observed in *Arabidopsis thaliana* and rice. The LRZ was analyzed using an Arizona microarray, which provided abundant candidate genes with diverse functions that are postulated to play important roles in adaptation to low-phosphate conditions. These findings enrich our understanding of plant responses to low phosphate levels and root morphogenesis.

## Methods

### Plant material

Seeds of the maize inbred line Q319 were surface-sterilized and placed on humid filter paper in sterile culture flasks held at 28 °C in darkness. The resulting seedlings (4 days after germination) were transferred from the culture flasks to a sufficient-phosphate (SP, 1,000 μM KH_2_PO_4_) solution (2 mM Ca(NO_3_)_2_.4H_2_O, 1.25 mM NH_4_NO_3_, 0.1 mM KCl, 0.65 mM K_2_SO_4_, 0.65 mM MgSO_4_, 10.0 mM H_3_BO_3_, 0.5 mM (NH_4_)_6_Mo_7_O2_4_, 1.0 mM MnSO_4_, 0.1 mM CuSO_4_.5H_2_O, 1.0 mM ZnSO_4_.7H_2_O, and 0.1 mM Fe-EDTA) and allowed to grow for 4 days (plants with 2–3 leaves), after which the endosperm was carefully removed. After 2–3 days of re-culturing in SP nutrient solutions, half of the seedlings were transplanted into a low-phosphate (LP, same composition as the SP solution, except that 5 μM KH_2_PO_4_ and 1 mM KH_2_PO_4_ were replaced with 1 mM KCl) nutrient solution. The plants were grown under a 32 °C/25 °C (day/night) temperature regime at a photon flux density (PFD) of 700 μmol m^-2^ s^-1^ with a 14-h/10-h light/dark cycle in a greenhouse with approximately 65 % relative humidity. The roots and leaves were harvested at 2 days and 8 days at 9:00–10:00 pm to avoid disturbing the circadian clock, respectively, after transfer into the SP/LP nutrient solutions for microarray and real-time RT-PCR analyses. The plants were subjected to measurements of morphological parameters and Pi content on days 1, 3, 6, 8 and 10 after treatment was initiated. According to this schedule, the time immediately before the seedlings was transferred into the LP nutrient solution was designated as 0 h.

### Measurement of root morphological and physiological parameters

The plant roots were scanned using a scanner (Powerlook 1000, China) and analyzed using LA-S-type plant image analysis software (developed at Zhejiang University, China). The numbers of axile and lateral roots were counted, and the total root length was measured. Feulgen staining was used to determine the number and density of lateral root primordia. The cell morphology of the elongation zone in the maize roots was analyzed by staining paraffin sections with hematoxylin.

Roots and shoots were dried in an oven at 80 °C to a constant weight and then weighed. The phosphate efficiency is expressed as the biomass (dry weight, g) produced by 100 mg of P. The P concentrations in roots and shoots were determined as described by Murphy and Riley [46].

### Microarray hybridization

The total RNA was extracted using the method of McCarty (1986) and purified using Qiagen RNeasy MinElute columns and buffers (Qiagen Cat # 74204). Then, 1.5 μg of the purified RNA was reverse-transcribed, amplified and labeled using Cy5 or Cy3 (Cy3/5™ Mono-Reactive Dye Pack, Cat#PA23001) with the Aminoallyl Message Amp II kit (Ambion, Cat# 1753) according to the manufacturer’s instructions. Unlabeled primers and dyes were removed using Qiagen RNeasy MinElute columns and buffers (Qiagen Cat # 74204). The labeled aRNA was then quantified, and the FOI was calculated.

Maize oligonucleotide array 46 K version slides were obtained from the microarray laboratory of the Maize Oligonucleotide Array Project at the University of Arizona. Four micrograms of mixed labeled aRNA was used in for microarray hybridization. The hybridizations were performed according to the protocols available at the website of the Maize Oligonucleotide Array Project (http://www.maizearray.org/). Following washing, the slides were immediately scanned using a LuxScan-10KA (SN100-0094, CapitalBio Corporation, China). The microarray used in this study contained 46,000 maize 70-mer oligonucleotides designated by TIGR ID, and the sequence information is available at the website of the Maize Oligonucleotide Array Project as the search item representing the >30,000 identifiable unique maize genes (details at http://www.maizearray.org). More than three independent experiments were performed for each biological sample derived from two independent treatments.

### Data export and analysis

Data acquisition and analysis were performed using a GenePix 4000B scanner with the GENEPIX 6.0 software (AXON INSTRUMENTS INC., USA). The overall intensity of the hybridized slide was normalized using GENEPIX 6.0. Spots flagged as *Bad* or *Not Found* by GENEPIX were removed from further data analysis, and only spots that showed fluorescence intensity levels above the 1.5-fold background level (local) in each channel were output by the software for further analysis. The GPR files were converted to the MEV format and LOWISS normalized using MADIS of TM4. Normalization of cross-slides was performed with Excel based on the average fluorescence signaling intensity of each slide. Then, a *t*-test was performed, and three well duplicates were selected to identify differentially expressed genes. In the present study, only transcripts with a stressed/control ratio ≤0.66 or ≥ 1.5 and a p value ≤0.05 were considered to be differentially expressed. The differentially expressed genes were annotated and classified using EasyGo (http://bioinformatics.cau.edu.cn/easygo/). To remove redundant transcripts, we used the following criteria: (1) transcripts with their top hit in the non-redundant amino acid database (NRAA), and (2) transcripts in the same contig using the CAP3 DNA sequence analysis program.

### Real-time RT-PCR of candidate genes

The total RNA was extracted from the roots after 2 days and 8 days of culture using the TRIzol reagent and then treated with RNase-free DNase. Next, cDNA synthesis was performed with the RT reagent kit (TAKARA, China) according to the manufacturer’s protocol. Real-time quantitative RT-PCR was performed in a Chromo^TM^ 4 (MJ Research, USA) using the SYBR Green RT-PCR Kit (Takara, China) and 10 μL reaction volumes, which contained 5 μL of SYBR Green PCR mix, 0.2 μM of each forward and reverse primer, 1 μL of diluted cDNA template, and the appropriate amount of sterile ddH_2_O to achieve the desired reaction volume. The amplification conditions were as follows: 2 min at 95 °C followed by 40 cycles of 15 s at 95 °C, 30 s at 58 °C, and 30 s at 72 °C. The levels of gene transcripts were calculated with the 2^-ΔΔCt^ method [47] using maize *Actin1* (NM_001155179.1) as an internal control due to the constitutive expression of this gene during the low-phosphate treatment. The entire experiment was repeated three times, and the primer sequences are shown in Additional file 2, Additional file 3 and Additional file 4.

## Abbreviations

LRZ, Lateral Root Primordium Zone; SP, Sufficient phosphate; LP, low phosphate; R/L, The root to shoot ratio; LOB, Lateral ogan bundaries; ARRs, Response Regulators; ABA, Abscisic acid.

## Authors’ contributions

ZL and CX performed the experiments and microarray data analysis. KL and CX analyzed the root morphology. SY and XQ assisted in data analysis. JZ contributed to the design of the experiments. ZL wrote the manuscript. All authors read and approved the final manuscript.

## Supplementary Material

Additional file 1 Differentially expressed genes identified in microarrayarrays. Click here for file

Additional file 2 Primers used for Real-time RT-PCR validation and expression analysis. Click here for file

Additional file 3 Table S2.Real-time RT-PCR to validate the results in the microarray analysis. Click here for file

Additional file 4 Expression analysis of some genes not significantly differentially expressed in microarray. Click here for file
