# Supplementary material for: Phosphate starvation of maize inhibits lateral root formation and alters gene expression in the lateral root primordium zone
Source: BMC Plant Biol. 2012 Jun 14;12:89. doi: 10.1186/1471-2229-12-89 (PMC3463438; doi:10.1186/1471-2229-12-89)
Supplement: Additional file 1 — Differentially expressed genes identified in microarrayarrays. [file 1471-2229-12-89-S1.doc]

# Additional files

**Additional file 1 Primers used for Real-time RT-PCR validation and expression analysis**

| Tigr_ID | Genebank_ID | Putative_Annotation | Sense primer | Antisense primer |
| --- | --- | --- | --- | --- |
| TM00018226 | U50064.1 | cyclin A-like protein CYCZM2W - maize {Zea mays;} | CATCACTAATCGCTGCCTCA | CAGGACCAACACTGGAAAGG |
| TM00043604 | NM_001154506 | putative cdc21 protein {Oryza sativa (japonica cultivar-group);} | GCATGTTACACGAGGTGATG | TTTCTGCTCCCAGGGCTATT |
| TM00017440 | EU944712.1 | protein kinase cdc2 homolog - rice {Oryza sativa;} | AAAGAAGTACACGCACGAGAT | CCAGGGACAAGAGTGGACAG |
| TM00015724 | EU972675.1 | G1/S-specific cyclin C-type. {Oryza sativa;} | GCACAGCAGGTTAAAGTTAG | CAATCATGTAGGGAGGGTAT |
| TM00026989 | BT034210.1 | putative cyclin-dependent kinase CDC2C {Oryza sativa (japonica cultivar-group);} | CTAGAGCGTCCAGATTCGT | TTCCTCACTTGTCCACCTT |
| TM00027288 | EU967414.1 | CDC2+/CDC28-related protein kinase R2 (EC 2.7.1.-). {Oryza sativa;} | TTGGATACGCGGAGTAGAAG | GTACAAACACGCATGGAGCA |
| TM00041533 | U87949.1 | proliferating cell nuclear antigen {Zea mays;} | ATGTTGGAGTTGCGTCTGG | AGGTGCTCGCTATCAATGTC |
| TM00027996 | NM_001130120.1 | putative tryptophan synthase alpha chain {Oryza sativa (japonica cultivar-group);} | CTATTGACAACACCGACTACACC | ACCTTGCCGCTTACATTTGC |
| TM00032240 | NM_001159145.1 | putative indole-3-glycerol phosphate synthase {Oryza sativa (japonica cultivar-group);} | ACCGCAGTCTTGGGACATTC | ACTCGCTCACAACCTGTATTCG |
| TM00014943 | NM_001112343 | anthranilate synthase alpha 2 subunit {Oryza sativa (japonica cultivar-group);} | CCAGAGTTGGGATGCCTTGA | GCCTATCTGCGTCTTTGTATGAG |
| TM00043903 | BT019285 | putative anthranilate phosphoribosyltransferase {Oryza sativa (japonica cultivar-group);} | GGCAGGATAAGGTCTGGATA | CAAGTCTTCTCAGGGTTCATT |
| TM00030640 | EU976463.1 | shikimate kinase {Oryza sativa (japonica cultivar-group);} | CTGCAAGAGCCTTGGGATG | AGCGGAACAACGGGAGAAT |
| TM00015709 | NM_001158928 | shikimate kinase {Oryza sativa (japonica cultivar-group);} | AGGCGTATTGCTAAAGTGGG | ATGTGGCAAACAAAGGGATT |
| TM00030748 | CF630212.1 | putative LOB domain protein 17 {Oryza sativa (japonica cultivar-group);} | TGCCATTGTTGGTTGGTAAA | CAAGGATGTGGAGATGGGTC |
| TM00042027 | AY359573.1 | acc oxidase {Zea mays;} | TTCTACAACCCAGCCAACGA | CGCACGGACACTCACCTATT |
| TM00016033 | DQ244273.1 | putative ethylene-responsive element binding factor {Oryza sativa (japonica cultivar-group);} | CAAGACCAACTTCTCCTTCCCTG | TCGGTGGCAGCTTCAGCAT |
| TM00025165 | EU962043.1 | putative ethylene-responsive small GTP-binding protein {Oryza sativa (japonica cultivar-group);} | CACCACCATTGGGATTGAC | CCCTCTTGAAGTTGGCACA |
| TM00033475 | DR906579.1 | beta-D-glucosidase (EC 3.2.1.-) glu2 precursor - maize {Zea mays;} | AGGCTACTTCGCTTGGTCTC | GAATCTTCTTGCTGGGCTTT |
| TM00036348 | BT056120.1 | response regulator 4 {Zea mays;} | CGAGGAGGCTGTCTACGAG | CCTTCCGAACGATTAACGA |
| TM00018488 | BT037683.1 | response regulator 7 {Zea mays;} | CTGGAGGGAAGAGGATGGG | TGAGCACGATGTCAATGGTTAT |
| TM00018690 | EU969358.1 | gibberellin 20-dioxygenase (EC 1.14.11.-) (clone S39A) [similarity] - wheat {Triticum aestivum;} | GACATCCCGCAGCAGTTCATC | AACGACAGCGTCTCCTTCCAG |
| TM00024317 | EU960670.1 | putative gibberellin induced protein 3 {Oryza sativa (japonica cultivar-group);} | CCTTTCCCGTGTCTGCTCT | GCCTTCCTGTACTGCGTCTT |
| TM00013510 | BT068939.1 | ZmGR1b {Zea mays;} | AAGGCGAGGCTGGCAAGGT | TGGCTGGAATGGTTCTAGGTT |
| TM00031932 | AJ278666.1 | putative Rop family GTPase ROP5 {Zea mays;} | AAAGAAAGAACGGCGAGGAT | GAGCCAATGGTACATAAACGAA |
| TM00017643 | AF376054.1 | putative Rop family GTPase ROP8 {Zea mays;} | TAATCCAGCCTCCAACTAAA | GACATTGCCAAGAAAGAACA |
| TM00024716 | AY110881.1 | GTPase activating protein-like {Oryza sativa (japonica cultivar-group);} | GGTGGCAACAAATGGAAAC | ATTGGATCGCTTGACAGGA |
| TM00014333 | FL468243.1 | putative GTPase activating protein {Oryza sativa (japonica cultivar-group);} | ACAGATGTACTAAGCCTCAACA | ACGAATAGCAGGTAACAGAAT |
| TM00004129 | EU963644.1 | SPX domain containing protein | TAGAAGCAAAGGGAAGCGAAGC | CACCATGACGCCGTGGAAG |
| TM00003825 | DQ468654 | bHLH transcription factor PTF1 {Oryza sativa;} | TCAACTTCTGTCAGCTCCAA | GCAGCAGCATGTCTTTATTT |
| TM00003660 | NM_001254768.1 | putative 3(2),5-bisphosphate nucleotidase {Oryza sativa (japonica cultivar-group);} | ATGGTCTAGGCGTCTGTCTG | CATGTTTGGCTATCTGGAATG |
| TM00003889 | NM_001177114.1 | probable myb-related protein - rice {Oryza sativa;} | TCCACCAGGGACGAGGACATG | CCCGACCGAGTTTCTCAGCATTT |
| TM00005265 | EU955623.1 | putative helix-loop-helix DNA-binding protein {Oryza sativa (japonica cultivar-group);} | CGTCCCTGGCTCTATTGATTT | AGGCGTCACCATTTGTAGCAG |
| TM00005295 | BT066873.1 | putative glucose inhibited division protein A {Oryza sativa (japonica cultivar-group);} | ACACGGCGTTGTATTCTCCC | CTTCGCCACTGATTTCCTTT |
| TM00007049 | EU972078.1 | putative RING3 protein {Oryza sativa (japonica cultivar-group);} | TGGCACTGTCCACTTTCTGA | ACACGCTGTCCTCCATTTCA |
| TM00013510 | BT068939.1 | ZmGR1b {Zea mays;} | AAGGCGAGGCTGGCAAGGT | TGGCTGGAATGGTTCTAGGTT |
| TM00013696 | NM_001154552.1 | putative 60S ribosomal protein L18a {Oryza sativa (japonica cultivar-group);} | CAAGACTGCGACGGTCCACT | TGGTCCTGAGCTTCCTGGTG |
| TM00015224 | EU975731.1 | putative zinc finger transcription factor {Oryza sativa (japonica cultivar-group);} | CGGCTTGGTATTTCTGTGAT | CTGTAGGGAATGCTGGGTCT |
| TM00015724 | EU972675.1 | G1/S-specific cyclin C-type. {Oryza sativa;} | GCACAGCAGGTTAAAGTTAG | CAATCATGTAGGGAGGGTAT |
| TM00015894 | L08426.1 | Auxin-binding protein 4 precursor (ABP). {Zea mays;} | TAATCTAATCACCGTGGCG | GCATCCAAGTGGCAGTAAG |
| TM00016294 | NM_001136737.1 | expansin EXPA7 {Triticum aestivum;} | TTGGGAGTTCGGCAAGACAT | GAGCCCTGCACTAAGTGATTTT |
| TM00016876 | NM_001196659.1 | putative cell division protein FtsH3 {Oryza sativa (japonica cultivar-group);} | CTGTCAAATGGTGGTTTAGATG | TAGCACGAATAACTGAAAGAGC |
| TM00017440 | EU944712.1 | protein kinase cdc2 homolog - rice {Oryza sativa;} | AAAGAAGTACACGCACGAGAT | CCAGGGACAAGAGTGGACAG |
| TM00018352 | EU969816.1 | putative OsGAI {Oryza sativa (japonica cultivar-group);} | GAGGCGGGTCCCTACCTCAAGTT | GGAGGCCCTGCATGATGCTGAA |
| TM00018488 | BT037683.1 | response regulator 7 {Zea mays;} | CTGGAGGGAAGAGGATGGG | TGAGCACGATGTCAATGGTTAT |
| TM00018542 | EU955981.1 | Putative EREBP-like protein {Oryza sativa (japonica cultivar-group);} | GCTGTTCGACTCGCTCCTCTTC | CATTCAGCTCACCATCGGCATC |
| TM00018690 | EU969358.1 | gibberellin 20-dioxygenase (EC 1.14.11.-) (clone S39A) [similarity] - wheat {Triticum aestivum;} | GACATCCCGCAGCAGTTCATC | AACGACAGCGTCTCCTTCCAG |
| TM00019815 | EU964332.1 | MFP1 attachment factor 1 {Zea mays;} | TGGCTTGGGTCTGAGTTTGTATG | TGGCTTGAAAGATGGGAGGAA |
| TM00019908 | EU952923.1 | similar to Lycopersicon esculentum putative acid phosphatase unknown protein {Oryza sativa (japonica cultivar-group);} | CAGCGACAACTGGGTGGTGGA | TGGGAGAAGCAGGCGAGCAG |
| TM00022691 | NM_001157684.1 | similar to Lycopersicon esculentum putative acid phosphatase unknown protein {Oryza sativa (japonica cultivar-group);} | CAACAAAGCACCGCAGCTACTAAA | GGTGTCCATGAGCGTGTTCCAG |
| TM00023811 | EU958664.1 | putative ribosomal protein L27a {Oryza sativa (japonica cultivar-group);} | AGTCCTTATTCGCCTCTTGGTT | TAGCACAGTTGATAGCGCAGTTA |
| TM00024317 | EU960670.1 | putative gibberellin induced protein 3 {Oryza sativa (japonica cultivar-group);} | CCTTTCCCGTGTCTGCTCT | GCCTTCCTGTACTGCGTCTT |
| TM00024713 | AY105006.1 | unknown protein {Oryza sativa (japonica cultivar-group);} | AAGAAGAACCGTATCGTGCCG | CGCTGCGTTTGTTTACATCCC |
| TM00024716 | AY110881.1 | GTPase activating protein-like {Oryza sativa (japonica cultivar-group);} | GGTGGCAACAAATGGAAAC | ATTGGATCGCTTGACAGGA |
| TM00024895 | BT036010.1 | putative abscisic acid-induced protein {Oryza sativa (japonica cultivar-group);} | TGTGAACCGCCGAATAGTGA | ACCGCTTTGTGCTTCTTGAT |
| TM00025165 | EU962043.1 | putative ethylene-responsive small GTP-binding protein {Oryza sativa (japonica cultivar-group);} | CACCACCATTGGGATTGAC | CCCTCTTGAAGTTGGCACA |
| TM00025532 | BT039131.1 | myb-related transcription factor MYB59 [imported] - Arabidopsis thaliana {Arabidopsis thaliana;} | GGTAGGAGTGAGCAGCGTTAG | GCCAGGGTGGAGGTAGTTG |
| TM00025945 | BT066761.1 | putative 1-aminocyclopropane-1-carboxylic acid(ACC) oxidase {Oryza sativa (japonica cultivar-group);} | GCAGGCTCTTCGTCAACATCG | CGCCCTTAAACCGCAACAAT |
| TM00026470 | EU974350.1 | putative trehalose-6-phosphate phosphatase {Oryza sativa (japonica cultivar-group);} | TTGACTATGACGGAACACTT | CACATCAGTTGACCGAATA |
| TM00026518 | NM_001176411.1 | putative Serine/threonine protein phosphatase BSL1 {Oryza sativa (japonica cultivar-group);} | TCAGTTCCCTGGTAATCCTT | GTCCACTGGTATCGCAATCA |
| TM00026989 | BT034210.1 | putative cyclin-dependent kinase CDC2C {Oryza sativa (japonica cultivar-group);} | CTAGAGCGTCCAGATTCGT | TTCCTCACTTGTCCACCTT |
| TM00027239 | NM_001157018.1 | putative ZIP-like zinc transporter {Oryza sativa (japonica cultivar-group);} | CTTTCAGCAGTTCTAATGGCATCT | ATATTTCATCAAGGGCAGGTCTC |
| TM00027395 | EU967454.1 | putative AP2-domain DNA-binding protein {Oryza sativa (japonica cultivar-group);} | TACTGACGGAAGCGAAGACCA | CCGTAGAACTCCCTGATGCTG |
| TM00027482 | EU967404.1 | C2 domain-containing protein-like {Oryza sativa (japonica cultivar-group);} | GTGGACATCCGCCCATTAG | CTCCTCGGCTAGGCAGTTC |
| TM00029159 | EU948182.1 | PHD finger protein-like {Oryza sativa (japonica cultivar-group);} | AACAGCCGTTGCCTCCACCT | CCTCCATGCCATACCACTCG |
| TM00032240 | EU976463.1 | putative indole-3-glycerol phosphate synthase {Oryza sativa (japonica cultivar-group);} | CTGCAAGAGCCTTGGGATG | AGCGGAACAACGGGAGAAT |
| TM00036217 | XM_002465809.1 | Ubiquitin-conjugating enzyme E2-21 kDa 2 (EC 6.3.2.19) (Ubiquitin-protein ligase 5) (Ubiquitin carrier protein 5). {Arabidopsis thaliana;} | TGACTCGGGTGATGAGGAGA | CATTCAGACTGCACGGGAAC |
| TM00038231 | EU973856.1 | Histone H4 homologue {Sesbania rostrata;} | AACATCCAGGGCATCACCAA | CAGAAATACAATCACCCACAGACA |
| TM00040160 | NM_001196663.1 | 60S ribosomal protein L10a-1. {Arabidopsis thaliana;} | ACAAAGGCTACAGTTAAGTTCC | TTCCCATAGTGCTCTTCAGG |
| TM00041130 | JN132820.1 | ubiquitin-conjugating enzyme E2 - maize {Zea mays;} | GAAATGCTGTGAAGGAGGAA | AGACCCAACACCACTATACGA |
| TM00041374 | EU961342.1 | histone H2A {Oryza sativa (japonica cultivar-group);} | CAGCGAGGGATAACAAGAAGACC | GCCGACTAACTGCGTGCTAAAC |
| TM00041715 | BT016689.1 | putative ubiquitin fusion-degradation protein {Arabidopsis thaliana;} | AGTATTATTGAGACCGATTGTGAG | CTTCTTTCTTGGGCTCCTCT |
| TM00042027 | AY359573.1 | acc oxidase {Zea mays;}(yiyou ) | TTCTACAACCCAGCCAACGA | CGCACGGACACTCACCTATT |
| TM00042491 | AY107108.1 | putative bZIP transcription factor RF2b {Oryza sativa (japonica cultivar-group);} | CTTCTCACCGTCGGCAACA | CAGCAAACAAACAAGCCAACAC |
| TM00042817 | BT065466.1 | histone H3 (clone pH3c-1) - alfalfa {Medicago sativa;} | TTGATGTTTCGTCCGCTTCG | GTCCTTGGGCATGATGGTGA |
| TM00043095 | BT085731.1 | Soluble inorganic pyrophosphatase (EC 3.6.1.1) (Pyrophosphate phospho-hydrolase) (PPase). {Zea mays;} | CCTGTTGTTCCTGGTTCGTT | GCTCGCTGATGTCGTTGTAG |
| TM00043626 | BT065002.1 | 26S proteasome regulatory particle triple-A ATPase subunit5a {Oryza sativa (japonica cultivar-group);} | CAGGAACTGGCAAGACACTC | CACCGCTAACTTCACTATCAA |
| TM00044307 | EU960857.1 | beta-expansin 8 {Zea mays;} | GCCCAACACCTTCTACCGC | TACGCCTGCCTCCTCCATT |
| TM00051931 | NM_001157511.1 | myb family transcription factor-like {Oryza sativa (japonica cultivar-group);} | CAATGGATGGCTTCCTGACG | TCTTTGCCTGCTGCTCTTCG |
| TM00052139 | NM_001154467.1 | scarecrow transcriptional regulator-like protein {Oryza sativa (japonica cultivar-group);} | TGGAGCGGTGCGAGGTGTTC | GGACCTAGCCGTCCCTTTACGC |
| TM00052234 | BT065676.1 | putative transcription factor {Oryza sativa (japonica cultivar-group);} | GTAAACTCTACCCGCCGTCTC | CTCTTGGCTTGGGCACATT |
| TM00057236 | EU963406.1 | putative NAC-domain protein {Oryza sativa (japonica cultivar-group);} | TAGGTTGGATGACTGGGTGCTG | GCCCTGGATGTCGTCGTAGC |
