# Supplementary material for: Phosphate starvation of maize inhibits lateral root formation and alters gene expression in the lateral root primordium zone
Source: BMC Plant Biol. 2012 Jun 14;12:89. doi: 10.1186/1471-2229-12-89 (PMC3463438; doi:10.1186/1471-2229-12-89)
Supplement: Additional file 2 — Primers used for Real-time RT-PCR validation and expression analysis. [file 1471-2229-12-89-S2.doc]

# Additional files

### Additional file 2 –Real-time RT-PCR to validate the results in the microarray analysis

| Tigr_ID | Genebank_ID | Putative_Annotation | Array | | qPCR | |
| --- | --- | --- | --- | --- | --- | --- |
|  |  |  | 2d ZLR | 8d ZLR | 2d ZLR | 8d ZLR |
| TM00018226 | U50064.1 | cyclin A-like protein CYCZM2W - maize {Zea mays;} | 0.89±0.23 | 0.48±0.05 | 0.93±0.23 | 0.32±0.05 |
| TM00043604 | NM_001154506 | putative cdc21 protein {Oryza sativa (japonica cultivar-group);} | 0.64±0.12 | 0.43±0.12 | 0.58±0.06 | 0.41±0.12 |
| TM00017440 | EU944712.1 | protein kinase cdc2 homolog - rice {Oryza sativa;} | 0.86±0.14 | 0.42±0.06 | 0.76±0.06 | 0.44±0.06 |
| TM00015724 | EU972675.1 | G1/S-specific cyclin C-type. {Oryza sativa;} | 1.01±0.10 | 1.56±0.09 | 1.09±0.08 | 1.76±0.03 |
| TM00026989 | BT034210.1 | putative cyclin-dependent kinase CDC2C {Oryza sativa (japonica cultivar-group);} | 0.87±0.23 | 1.70±0.10 | 0.97±0.13 | 1.90±0.10 |
| TM00027288 | EU967414.1 | CDC2+/CDC28-related protein kinase R2 (EC 2.7.1.-). {Oryza sativa;} | 1.36±0.09 | 1.65±0.06 | 1.16±0.09 | 1.85±0.06 |
| TM00041533 | U87949.1 | proliferating cell nuclear antigen {Zea mays;} | 0.69±0.17 | 0.38±0.03 | 0.59±0.12 | 0.32±0.03 |
| TM00027996 | NM_001130120.1 | putative tryptophan synthase alpha chain {Oryza sativa (japonica cultivar-group);} | 0.79±0.09 | 1.94±0.08 | 0.69±0.09 | 2.21±0.08 |
| TM00032240 | NM_001159145.1 | putative indole-3-glycerol phosphate synthase {Oryza sativa (japonica cultivar-group);} | 1.38±0.15 | 3.09±0.05 | 1.18±0.15 | 3.42±0.05 |
| TM00014943 | NM_001112343 | anthranilate synthase alpha 2 subunit {Oryza sativa (japonica cultivar-group);} | 0.94±0.16 | 1.64±0.11 | 0.84±0.12 | 1.74±0.11 |
| TM00043903 | BT019285 | putative anthranilate phosphoribosyltransferase {Oryza sativa (japonica cultivar-group);} | 1.62±0.04 | 0.88±0.23 | 1.82±0.08 | 0.89±0.23 |
| TM00030640 | EU976463.1 | shikimate kinase {Oryza sativa (japonica cultivar-group);} | 1.22±0.22 | 1.81±0.07 | 1.12±0.02 | 1.89±0.07 |
| TM00015709 | NM_001158928 | shikimate kinase {Oryza sativa (japonica cultivar-group);} | 1.17±0.01 | 2.02±0.05 | 1.37±0.12 | 2.52±0.05 |
| TM00030748 | CF630212.1 | putative LOB domain protein 17 {Oryza sativa (japonica cultivar-group);} | 1.98±0.20 | 4.21±0.12 | 1.68±0.03 | 4.81±0.12 |
| TM00042027 | AY359573.1 | acc oxidase {Zea mays;} | 1.07±0.23 | 1.73±0.04 | 0.87±0.03 | 1.93±0.04 |
| TM00016033 | DQ244273.1 | putative ethylene-responsive element binding factor {Oryza sativa (japonica cultivar-group);} | 0.88±0.07 | 0.44±0.09 | 0.78±0.08 | 0.40±0.09 |
| TM00025165 | EU962043.1 | putative ethylene-responsive small GTP-binding protein {Oryza sativa (japonica cultivar-group);} | 0.90±0.09 | 1.59±0.11 | 0.96±0.10 | 1.89±0.11 |
| TM00033475 | DR906579.1 | beta-D-glucosidase (EC 3.2.1.-) glu2 precursor - maize {Zea mays;} | 2.06±0.04 | 0.67±0.04 | 2.56±0.04 | 0.67±0.04 |
| TM00036348 | BT056120.1 | response regulator 4 {Zea mays;} | 1.68±0.13 | 1.03±0.07 | 1.98±0.03 | 1.43±0.07 |
| TM00018488 | BT037683.1 | response regulator 7 {Zea mays;} | 0.82±0.08 | 0.56±0.08 | 0.92±0.06 | 0.36±0.45 |
| TM00018690 | EU969358.1 | gibberellin 20-dioxygenase (EC 1.14.11.-) (clone S39A) [similarity] - wheat {Triticum aestivum;} | 1.53±0.14 | 1.32±0.02 | 1.63±0.12 | 1.22±0.02 |
| TM00024317 | EU960670.1 | putative gibberellin induced protein 3 {Oryza sativa (japonica cultivar-group);} | 0.69±0.12 | 0.59±0.07 | 0.67±0.05 | 0.34±0.07 |
| TM00013510 | BT068939.1 | ZmGR1b {Zea mays;} | 0.37±0.22 | 0.36±0.09 | 0.27±0.02 | 0.36±0.04 |
| TM00031932 | AJ278666.1 | putative Rop family GTPase ROP5 {Zea mays;} | - | 0.39±0.03 | 0.49±0.04 | 0.36±0.03 |
| TM00017643 | AF376054.1 | putative Rop family GTPase ROP8 {Zea mays;} | 0.58±0.06 | 1.07±0.06 | 0.68±0.02 | 1.12±0.03 |
| TM00024716 | AY110881.1 | GTPase activating protein-like {Oryza sativa (japonica cultivar-group);} | 1.46±0.20 | 2.32±0.08 | 1.63±0.03 | 2.52±0.08 |
| TM00014333 | FL468243.1 | putative GTPase activating protein {Oryza sativa (japonica cultivar-group);} | 1.24±0.42 | 2.19±0.04 | 1.44±0.12 | 2.49±0.04 |
| TM00004129 | EU963644.1 | SPX domain containing protein | 2.49±0.02 | 4.38±0.08 | 2.64±0.08 | 4.56±0.12 |
| TM00003825 | DQ468654 | bHLH transcription factor PTF1 {Oryza sativa;} | 0.92±0.32 | 1.9±0.04 | 1.34±0.12 | 2.21±0.07 |

**^*^** Values means that the ratio of signal intense (LP treated/SPcontrol)
